# Evaluating the accuracy and reliability of telephone‐derived Clinical Dementia Rating scores: A comparative analysis with in‐person assessments

**DOI:** 10.1002/alz.71601

**Published:** 2026-06-18

**Authors:** Araya Dimtsu Assfaw, Chengjie Xiong, Krista L. Moulder, John C. Morris

**Affiliations:** ^1^ Department of Neurology Washington University School of Medicine in St. Louis St. Louis Missouri USA; ^2^ Knight Alzheimer Disease Research Center Washington University School of Medicine in St. Louis St. Louis Missouri USA; ^3^ Division of Biostatistics Washington University in St. Louis St. Louis Missouri USA

**Keywords:** Alzheimer disease and related dementias (ADRD), Clinical Dementia Rating (CDR), dementia staging, informant report, psychometrics, remote cognitive assessment, telephone assessment

## Abstract

**INTRODUCTION:**

The Clinical Dementia Rating (CDR) scale typically is administered in person, but use of telephone‐based, informant‐only assessments increased during the coronavirus disease 2019 (COVID‐19) pandemic. The correspondence of informant‐only assessments with in‐person ratings remains unclear.

**METHODS:**

We analyzed 1,140 paired in‐person and telephone assessments from the Knight Alzheimer's Disease Research Center Memory and Aging Project conducted within 12 months. Agreement for global CDR and CDR Sum of Boxes (CDR‐SB) was examined using kappa statistics, intraclass correlation coefficients (ICCs), and Bland–Altman methods; classification performance of telephone global CDR score ≥ 0.5 was assessed.

**RESULTS:**

Agreement for the global CDR was moderate (*κ =* 0.53, 95% CI: 0.47–0.59). CDR‐SB demonstrated moderate reliability (ICC = 0.69, 95% confidence interval [CI]: 0.65–0.73). Telephone CDR‐SB scores averaged 0.40 points higher than in‐person scores, with wide limits of agreement. Sensitivity was 64.2% and specificity 91.9%.

**DISCUSSION:**

Telephone CDR‐SB shows moderate concordance with in‐person CDR‐SB but shows consistent score inflation, which may limit clinical staging utility.

## Highlights

- Telephone Clinical Dementia Rating (CDR) showed moderate agreement with in‐person ratings
- Agreement was stable across demographics and assessment order
- Telephone CDR may support research but not replace clinical staging

## 1 BACKGROUND

Alzheimer disease and Alzheimer's disease‐related dementias (AD/ADRD) represent a growing global public health crisis, contributing substantially to morbidity and mortality among older adults. As the aging population increases, the prevalence of AD/ADRD is rising, driving significant investments in the development of effective treatments and interventions.^1^ Although recent advancements have led to the approval of disease‐modifying therapies such as lecanemab (Leqmbi®) and donanemab (Kisunla®), critical gaps remain in the timely and accurate diagnosis of AD/ADRD. Early and accurate cognitive assessment is essential to detect AD/ADRD at its symptomatic onset, when therapeutic strategies are likely to be most effective.^2^

The Clinical Dementia Rating (CDR) scale is a widely used and internationally validated instrument for staging dementia severity.^3^, ^4^ Administered through semi‐structured interviews with both a participant and a study partner (informant), the CDR evaluates current participant performance in comparison with the individual's previous levels of performance in six domains: Memory, Orientation, Judgment and Problem Solving, Community Affairs, Home and Hobbies, and Personal Care. A global CDR score is derived from the ratings of performance in these domains: 0 = normal, 0.5 = very mild dementia, 1 = mild dementia, 2 = moderate dementia, and 3 = severe dementia. Additionally, the CDR Sum of Boxes (CDR‐SB) is derived by summing the six individual domain box scores, yielding a continuous measure ranging from zero to eighteen.^5^ While the global CDR is an ordinal scale determined through an algorithm that integrates severity across domains, the CDR‐SB provides a more granular index of impairment and often demonstrates greater sensitivity to subtle cognitive and functional change. Thus, although related, the global CDR and CDR‐SB capture complementary aspects of dementia severity, with CDR‐SB offering finer differentiation within and across stages. CDR demonstrates strong inter‐rater reliability and validity across diverse settings and has been translated into more than 80 languages, underscoring its broad applicability in both research and clinical practice.^3^, ^4^, ^5^, ^6^, ^7^, ^8^, ^9^

**RESEARCH IN CONTEXT**

1. **Systematic review**: We reviewed published studies using PubMed and recent Alzheimer's Disease Research Center (ADRC) and National Alzheimer's Coordinating Center  (NACC) presentations to identify evidence on remote administration of the Clinical Dementia Rating (CDR) and related cognitive assessments. Existing work supports the feasibility of remote and informant‐based evaluations, but few studies have directly compared telephone versus in‐person CDR ratings. Prior findings are mixed, with limited sample sizes and minimal domain‐level evaluation.
2. **Interpretation**: Our results demonstrate that telephone‐administered CDRs show moderate agreement with in‐person assessments but exhibit systematic score inflation and wide individual‐level variability, particularly in CDR‐SB. These findings clarify the extent to which informant‐only telephone assessments align with the current gold standard and highlight important limitations.
3. **Future directions**: Future research should validate remote CDR protocols across the full severity spectrum (including CDR score ≥ 2), incorporate video or hybrid methods, evaluate strategies to adjust for modality‐related bias, and test remote assessments in more diverse and community‐based populations.

During the coronavirus disease 2019 (COVID‐19) pandemic and accompanying inability to conduct in‐person assessments, the Memory and Aging Project (MAP) Charles F. and Joanne Knight Alzheimer Disease Research Center (Knight ADRC) transitioned to telephone‐based CDR administration for its research participants using an informant‐only model. While initially a public health necessity, remote approaches remain valuable for improving access to cognitive assessments among rural, underserved, and mobility‐limited populations.^10^, ^11^ Importantly, the telephone CDR administration differs from the in‐person standard: the interview of the participant is omitted, and scoring relies exclusively on the informant reports. This change avoids potential bias from participant use of undetectable environmental cues such as calendars, clocks, or outside assistance to inform their responses, but may also reduce reliability for the CDR domains (Memory, Orientation, Judgment, and Problem Solving) that normally are based on both participant and informant input.

Although in‐person assessments remain the gold standard because they allow direct clinician interaction with both participant and informant,^12^ telephone‐based assessments offer several logistical advantages, including reduced participant burden, especially for those who are disabled or have more advanced dementia, efficient use of site resources, and greater retention in longitudinal studies through flexible follow‐up.^13^ Indeed, even post‐pandemic, approximately 2–5% of the CDR assessments at the Knight ADRC continue to be conducted remotely. Data from the National Alzheimer's Coordinating Center (NACC) show that 26 of 37 ADRCs have incorporated remote cognitive assessments, primarily via telephone,^14^ reflecting a broader shift toward integrating remote methodologies into dementia research.

Evidence supporting remote cognitive assessments is growing. Feasibility studies^15^, ^16^, ^17^, ^18^, ^19^, ^20^ and systematic reviews.^21^, ^22^, ^23^, ^24^generally support the reliability of remote tools. However, relatively few investigations have directly compared telephone‐administered versus in‐person CDR scores, and findings remain limited.^25^, ^26^ Additionally, artificial intelligence (AI) ‐assisted approaches, including voice‐based CDR administration, are emerging although as yet evidence is lacking to support their use as a substitute for in‐person administered CDR.

To address the underexplored knowledge gap regarding the reliability of remote CDR assessments, this study evaluated agreement between in‐person and telephone‐based CDR scores in Knight ADRC participants who had administration of each method within a 12‐month period.

## 2 METHODS

### 2.1 Study design and setting

**FIGURE 1:**
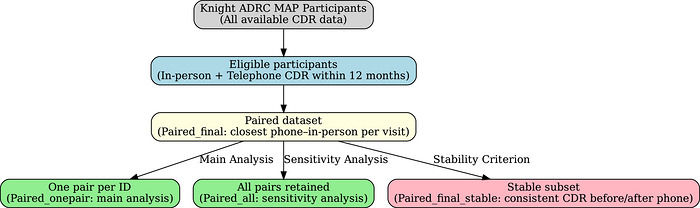
Flow of participant inclusion and analytic sample selection.

We conducted a retrospective, cross‐sectional validation study using secondary longitudinal data from the Knight ADRC. The Knight ADRC is based in St. Louis, Missouri, and enrolls a diverse cohort of older adults, typically including both cognitively unimpaired participants and individuals with very mild dementia, reflecting the sociodemographic and clinical characteristics of the regional population. Participants were eligible if they had both an in‐person CDR assessment and a telephone CDR completed within a 12‐month interval (≤ 365 days). We excluded records with incomplete CDR data. Participants with a global CDR ≥2 are not followed in the Knight ADRC because they are generally unable to complete the full research protocol, which includes extensive imaging and cerebrospinal fluid studies. A CONSORT‐style flow diagram (Figure 1) illustrates selection from the MAP cohort. The pairing procedure identified each telephone CDR assessment and selected the temporally nearest in‐person CDR assessment visit within a 12‐month window (≤ 365 days), allowing for visits occurring either before or after the telephone assessment. If multiple pairs existed, then they were included in the sensitivity analysis. The available data span 2020–2024.

### 2.2 Participant characteristics and data collection

Demographic variables (age, sex, race, ethnicity) were abstracted from study records. Clinical variables included the global CDR and the six domain box scores (Memory, Orientation, Judgment and Problem Solving, Community Affairs, Home and Hobbies, Personal Care). The CDR was computed by summing the domain scores. For each telephone assessment, we paired the temporally closest in‐person assessment within 12 months and recorded assessment order (telephone‐first vs in‐person‐first) and the inter‐visit interval (days/months). Because this retrospective dataset did not record clinician or informant identifiers, we could not determine whether the same clinician or study partner contributed to both the in‐person and telephone assessments. Therefore, agreement estimates reflect the combined influence of assessment modality, potential rater variability, and informant differences.

### 2.3 Statistical analyzes

Statistical analyzes were conducted in SAS 9.4 (SAS Institute, Cary, NC). Descriptive statistics were used to summarize demographic characteristics and CDR distributions. Agreement for categorical ratings (global CDR and domain boxes) was quantified using weighted kappa.^27^which is more appropriate for ordinal data such as the CDR. All weighted kappa and interclass correlation coefficients (ICC) estimates are reported with corresponding 95% confidence intervals (CIs). For continuous outcomes (CDR‐SB), reliability was assessed using ICC based on the Shrout–Fleiss ICC(3,1) formulation.^28^All kappa values were interpreted using conventional thresholds: < 0.20 poor, 0.21–0.40 fair, 0.41–0.60 moderate, 0.61–0.80 substantial, and > 0.80 near‐perfect agreement.^29^ICC values were interpreted using commonly applied benchmarks: < 0.50 poor, 0.50–0.75 moderate, 0.75–0.90 good, and > 0.90 excellent reliability.^30^To evaluate modality‐related differences in CDR‐SB, we fit a linear mixed‐effects model with a random intercept for participants and a fixed effect for modality (in‐person vs. telephone), an approach that accounts for within‐participant correlation in repeated assessments.^31^The analytic unit for this study was the paired CDR assessment. The dataset included 1,140 paired in‐person and telephone evaluations, and some participants contributed more than one paired observation within the specified time window. Accordingly, the reported sample size reflects paired assessments rather than unique participants.

### 2.4 Agreement, reliability, and bias

Bland–Altman analysis evaluated systematic bias (mean difference) and variability (95% limits of agreement) between telephone and in‐person CDR‐SB scores, with visualization and interpretation regarding clinical interchangeability. We interpreted Bland–Altman results by considering both mean bias and individual‐level limits of agreement, as small average differences may coexist with clinically meaningful variability

### 2.5 Classification performance and sensitivity/subgroup analyzes

Because the primary objective of this study was to evaluate agreement between telephone and standard in‐person CDR administration, the in‐person global CDR was treated as the reference comparator when evaluating the classification performance of telephone CDR at ≥ 0.5 (impaired) by calculating sensitivity, specificity, positive/negative predictive values, and accuracy from 2×2 tables. We examined agreement by assessment interval (≤ 3, 4–6, 7–12 months) and by sex, race, and education. Sensitivity analyzes included restrictions to one pair per participant and a stability subset. For stability analyzes, we required that the participants’ in‐person global CDR at the visits immediately preceding and following the telephone CDR were identical, to reduce the chance that true clinical change (rather than modality differences) drove disagreement. Because some participants contributed more than one in‐person/telephone pair, we conducted a parallel analysis restricted to one randomly selected pair per participant to avoid violation of independence assumptions; full‐sample results are presented as sensitivity analyzes. Although the primary analyzes dichotomized CDR at ≥ 0.5 to align with standard classifications of impairment, an alternative approach would be to present full 3×3 agreement tables for CDR 0, 0.5, and 1. However, given the study objective of distinguishing impaired versus unimpaired status, the dichotomized approach remains appropriate and interpretable for this analysis.

## 3 RESULTS

### 3.1 Participant characteristics

A total of 1,140 paired in‐person and telephone CDR assessments conducted within a 12‐month interval were included. The closest in‐person CDR assessment within the 12‐month window occurred before the telephone CDR in 58.1% of pairs (*n =* 662), after the telephone CDR in 41.3% (*n =* 471), and on the same day in 0.6% (*n = *7), reflecting a bidirectional temporal distribution inherent to the nearest‐visit matching approach. Participants had a mean age of 73.9 years (SD = 8.1; range 49‐97); 58.3% were female, and 82.7% identified as White, while 16.6% were Black, 0.2% Asian, and 0.5% reported more than one race. Hispanic ethnicity was reported by 0.35% of participants. Table 1 summarizes the demographic characteristics of the cohort.

**TABLE 1:** Participant demographic and clinical characteristics (*N = *1,140 paired assessments)

Characteristic	Category	Value
**Sex**		
	Female	665 (58.33%)
	Male	475 (41.67%)
**Race**		
	Asian	2 (0.18)
	Black	189 (16.58%)
	White	943 (82.72%)
	More than one race	6 (0.53%)
**Ethncity**		
	Hispanic	4 (0.35)
	Not Hispanic	1130 (99.34%)
	Not identified	6 (0.53%)
**Age (years)**	N	1,140
	Mean (SD)	73.89 (8.12)
	Median (IQR)	74 (68–79)
	Range	49–97
**Education (Categorized)**		
	High School (≤12 years)	127 (11.14%)
	Some College (13–15 years)	193 (16.93%)
	Bachelor's degree (16 years)	290 (25.44)
	Graduate/Professional degree (≥ 17)	530 (46.49)
**APOE genotype**		
	ε2/ε2	5 (0.44%)
	ε2/ε3	102 (8.95)
	ε2/ε4	35 (3.07%)
	ε3/ε3	485 (42.54)
	ε3/ε4	259 (22.72)
	ε4/ε4	32 (2.81)
	Missing	222 (19.47)
**CDR‐Sum of Boxes (In‐Person)**	N	1,140
	Mean(SD)	0.46 (1.23)
	Median(IQR)	0(0‐0)
	Range	0‐9
**CDR‐Sum of Boxes (Telephone)**	N	1,140
	Mean(SD)	0.58 (1.52)
	Median(IQR)	0 (0‐0)
	Range	0‐9

Abbreviations: APOE, apolipoprotein E; CDR, Clinical Dementia Rating.

The CDR‐SB scores were higher for telephone (*M =* 0.58, *SD =* 1.52) compared with in‐person (*M =* 0.46, *SD =* 1.23) evaluations, suggesting a modest tendency for telephone assessments to yield slightly higher impairment ratings (Table 1).

### 3.2 Agreement on global CDR

The agreement between modalities was moderate. The weighted kappa was 0.53 (95% CI 0.47–0.59), below the conventional threshold for substantial agreement (> 0.60) as shown in Table 2 below.

**TABLE 2:** Kappa statistics.

Kappa statistics				
Statistic	Estimate	Standard error	95% Confidence limits	
Weighted kappa	0.5263	0.0303	0.4670	0.5856

Cross‐tabulation demonstrated strong concordance at the no impairment level (CDR = 0) but more disagreement at higher impairment levels (Table 3). Among participants rated CDR = 0.5 in person, ∼41% were scored as 0 and ∼51% as 0.5 by telephone. By assessment method, most participants were classified as CDR 0 (*n =* 939), with smaller proportions at CDR 0.5 (*n =* 166) and CDR 1 (*n =* 35) (see Table 3 for counts and percentages by modality).

**TABLE 3:** Cross‐Tabulation of global CDR ratings: In‐person vs. telephone assessments.

Global CDR (in‐person)	Global CDR (telephone)			
Frequency	0.0	0.5	1.0	Total
0.0	863	62	14	939
0.5	67	76	23	166
1.0	5	10	20	35
Total	935	148	57	1140

### 3.3 Domain‐level agreement

Agreement between modalities varied across CDR domains (Table 4). Weighted kappa statistics indicated moderate concordance for Home and Hobbies (*κ =* 0.52, 95% CI: 0.45–0.59) and Memory (*κ =* 0.51, 95% CI: 0.45–0.57), followed by Judgment and Problem Solving (*κ =* 0.49, 95% CI: 0.44–0.55), Orientation (*κ =* 0.47, 95% CI: 0.40–0.54), and Community Affairs (*κ = *0.46, 95% CI: 0.38–0.53). Personal Care showed the lowest agreement (*κ =* 0.42, 95% CI: 0.26–0.58), although estimates for this domain were less stable because relatively few participants required assistance with basic activities of daily living.

**TABLE 4:** Domain‐level agreement between in‐person and telephone CDRs.

Domain	Weighted kappa (95%)
Memory	0.51 (0.45–0.57)
Judgment and Problem Solving	0.49 (0.44–0.55)
*Orientation*	0.47 (0.40–0.54)
*Community Affairs*	0.46 (0.38–0.53)
*Home & Hobbies*	0.52 (0.45–0.59)
*Personal Care*	0.42 (0.26–0.58)*

^*^ Indicate that Personal Care estimates should be interpreted with caution becuase relatively few particpants required assistance with basic activities of daily living, resulting in less stable agreement estimates.

### 3.4 Reliability of CDR‐SB

The CDR‐SB showed moderate reliability. The intraclass correlation coefficient (Shrout–Fleiss ICC (3,1))^26^ was 0.69 (95% CI: 0.65–0.73), consistent with commonly applied ICC thresholds for moderate reliability (0.50–0.75). Mixed‐effects modeling^31^ confirmed a significant modality effect (F(1,1657) = 51.2, *p <* 0.0001), with telephone scores on average 0.40 points higher than in‐person.

### 3.5 Bland–Altman analysis

**FIGURE 2:**
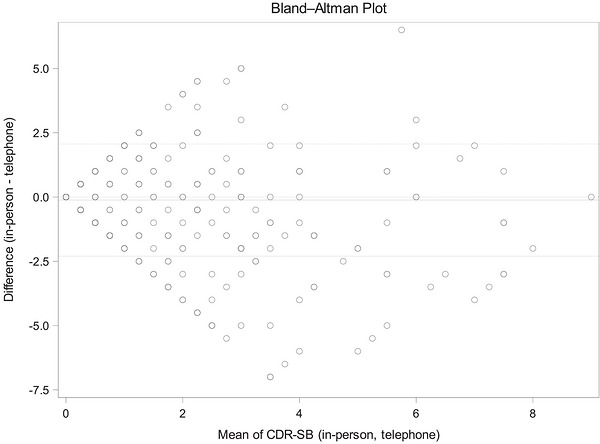
Bland–Altman plot comparing in‐person and telephone CDR‐SB scores. CDR‐SB, Clinical Dementia Rating Sum of Boxes.

The Bland–Altman analysis showed that the mean difference between in‐person and telephone CDR‐SB scores was small (–0.12; SD = 1.11), indicating minimal average bias between modalities (Figure 2). However, the 95% limits of agreement were wide (–2.30 to 2.06), demonstrating substantial variability at the individual level. Thus, while mean differences were negligible across the cohort, disagreement between modalities for specific participants was often large enough to alter clinical staging. Variability increased with higher mean CDR‐SB scores, suggesting that inconsistencies were more pronounced among individuals with greater impairment.

Telephone assessments systematically yielded higher scores (mean difference –0.40), with wide 95% limits of agreement (–4.15 to 3.35). Variability increased at higher mean CDR‐SB values, indicating that while group‐level trends align, individual‐level agreement is limited, particularly in more impaired participants.

### 3.6 Classification performance

Using in‐person global CDR ≥ 0.5 as the comparator reference, telephone‐based classification demonstrated good overall accuracy. Sensitivity was 64.2%, indicating that nearly two‐thirds of impaired cases were correctly identified, while specificity was 91.9%, reflecting a strong ability to rule out unimpaired cases. The positive predictive value was 62.9%, and the negative predictive value was 92.3%, underscoring that unimpaired classifications were more reliable than impaired ones. Overall accuracy was 87.0%. Misclassification clustered around the 0.5 threshold, where both false positives and false negatives were observed, highlighting the challenge of discriminating very mild impairment. This pattern reflects the clinical heterogeneity of the CDR 0.5 category and the concentration of the analytic sample within the earliest stages of cognitive impairment.

### 3.7 Sensitivity and subgroup analyzes

Sensitivity analyzes restricting the dataset to one randomly selected in‐person/telephone pair per participant yielded agreement estimates similar to the primary analysis (*k = *0.54, 95% CI: 0.45–0.62), indicating that repeated observations from the same individual did not materially influence the findings. Similarly, analyzes restricted to the stability subset showed moderate agreement (*k =* 0.48, 95% CI: 0.34–0.62). Agreement was highest when telephone and in‐person assessments occurred within ≤3 months (*k =* 0.53, 95% CI: 0.47–0.59), with modest attenuation observed at longer inter‐assessment intervals. Subgroup analyzes demonstrated similar agreement across sex and race strata, while participants with fewer than 16 years of education showed lower concordance. Overall, subgroup and sensitivity analyzes demonstrated that the primary findings were robust across analytic restrictions and assessment timing conditions. See Table S1.

## 4 DISCUSSION

This study evaluated the comparability and reliability of the telephone‐administered CDR scale against the traditional in‐person assessment using 1,140 paired evaluations from the Knight ADRC MAP. Overall, telephone‐based CDR ratings demonstrated moderate agreement with in‐person assessments and moderate‐to‐good reliability for the CDR Sum of Boxes (CDR‐SB). However, the telephone assessment showed slightly higher impairment ratings compared with in‐person assessments. Within the National Institute on Aging and Alzheimer's Association (NIA‐AA) framework for ADRD diagnosis, the CDR provides a functional assessment that complements biomarker and cognitive testing, helping to identify early clinical change.

The agreement between in‐person and telephone global CDR scores was moderate (*k =* 0.53, 95% CI: 0.47–0.59), falling within the conventional range for moderate agreement (0.41–0.60).^32^ These values are lower than the high inter‐rater reliability consistently reported for in‐person CDR assessments,^3^, ^6^underscoring the limitations of informant‐only protocols. Misclassification was most pronounced at the threshold between unimpaired and very mild impairment: approximately 41% of participants rated as CDR 0 in‐person were scored as 0.5 by telephone. This systematic inflation may reflect informant over‐reporting of cognitive symptoms, as informants may be more likely to attribute subtle lapses to impairment rather than normal variability or compensatory strategies.^33^

The CDR‐SB, as a continuous measure, provided a complementary view of cross‐modal agreement. However, mixed‐effects models revealed a consistent modality effect, with telephone assessments scoring on average 0.40 points higher than in‐person assessments. Bland–Altman analysis confirmed this systematic bias and demonstrated wide limits of agreement, indicating that discrepancies at the individual level could be clinically meaningful. The observed differences in CDR‐SB scores between assessment modalities were small relative to published estimates of clinically meaningful change for CDR‐SB, suggesting that any measurement variability is unlikely to represent clinically significant differences in functional staging. Similar findings of inflated impairment ratings in remote or informant‐only assessments have been reported in pandemic‐era studies across ADRCs.^15^, ^25^CDR scoring relies heavily on clinical judgment, including the depth of probing, interpretation of informant descriptions, and integration of information derived from the participants. Because clinical and informant identities were not available in the dataset, the observed agreement likely reflects a combination of modality effects and inter‐rater or informant variability rather than modality differences alone.

Domain‐specific analyzes further illustrate the consequences of omitting participant interviews in telephone administration. The agreement was moderate for Memory, Judgment, Orientation, Community Affairs, and Home and Hobbies. By contrast, Personal Care showed only fair agreement, a finding likely influenced by sparse data, as fewer participants in this sample required assistance with basic activities of daily living. Domains that depend on structured clinician–participant probing, including Memory, Judgment, and Orientation appear more vulnerable to measurement error when the participant interview is omitted^3^, ^34^. In contrast, domains reflecting overt functional decline, such as Personal Care, are more reliably reported by informants, though the limited variability in this domain likely constrained reliability estimates.

Using in‐person global CDR score ≥ 0.5 as the comparator reference, the telephone CDR demonstrated an overall accuracy of 87.0%. Specificity and negative predictive value were high, underscoring the ability of telephone assessments to reliably identify unimpaired individuals. However, sensitivity was more modest, with a positive predictive value, reflecting reduced capacity to detect very mild impairment. Importantly, misclassification clustered around the 0.5 threshold, the critical boundary between normal cognition and the earliest detectable impairment. At this cutpoint, both false positives and false negatives were observed. Another important consideration is the role of study partners, whose reports form the basis of telephone‐administered CDR ratings. Characteristics such as relationship to the participant, co‐residency, familiarity with daily functioning, and frequency of contact can meaningfully influence the accuracy and completeness of the information they provide. These factors may contribute to over‐reporting or under‐reporting of impairment, particularly in the absence of clinician–participant interaction.

Robustness checks confirmed the stability of the primary findings. Restricting the sample to one assessment pair per participant produced results nearly identical to the full dataset, supporting the reliability of our analytic approach. Temporal proximity between paired assessments significantly influenced agreement: concordance was highest when telephone and in‐person visits occurred within three months and declined modestly when the interval extended to 7–12 months. Subgroup analyzes further contextualized these findings. Agreement was broadly stable across sex and race, indicating that telephone assessments performed comparably in men and women and across White and Black participants. However, modest reductions in reliability were observed among participants with fewer years of education, which may reflect differences in informant quality or reporting style.^35^

These limitations underscore the need for caution when applying telephone CDRs in clinical care, especially for staging disease severity or tracking longitudinal change, where even small score differences can alter diagnostic or therapeutic decisions. In contrast, for research applications, particularly large‐scale longitudinal cohorts where participant retention, accessibility, and scalability are essential, telephone CDRs offer a pragmatic and cost‐effective solution.^10^, ^19^

Our results extend prior feasibility studies that demonstrated the practicality and acceptability of remote dementia assessments in both research and clinical contexts^10^, ^19^ ^,^ ^26^A previous study conducted informant‐based, including remote, CDR assessments, supporting our rationale for telephone evaluation.^36^Our findings also align with more recent evidence from the COVID‐19 pandemic, during which ADRCs and other institutions rapidly adopted telephone‐based CDR assessments to maintain continuity of longitudinal follow‐up.^15^, ^25^These pandemic‐era adaptations underscored the feasibility of scaling remote assessments across diverse sites, but they were often driven by necessity rather than by formal validation.

However, the moderate agreement observed here stands in contrast to the near‐perfect inter‐rater reliability historically reported for in‐person CDR administration.^3^, ^6^where κ values often exceed 0.80. This discrepancy highlights the unique value of clinician–participant interaction, which allows for nuanced probing, observation of compensatory strategies, and correction of reliance on external cues, elements that are inherently absent from informant‐only telephone protocols.

Telephone‐administered CDR assessments provide a practical and moderately reliable complement to in‐person evaluation, particularly when face‐to‐face assessment is not feasible, including in rural, mobility‐limited, or public health emergency contexts, and can support retention, inclusivity, and longitudinal follow‐up in research settings. However, their tendency to yield modestly higher scores, especially near the CDR 0.5 threshold, limits their interchangeability with in‐person assessments for clinical staging or diagnostic decision‐making, underscoring the need to interpret results alongside other clinical data and to follow up borderline cases. Limitations of the study include the retrospective design, potential non‐independence of repeated observations due to participants contributing to more than one paired assessment, including lack of informant‐level data, relationship with the participant, co‐residence, and frequency of interaction, which may all influence reporting accuracy, possible interval‐related clinical change, predominantly White study group, highly educated sample, and underrepresentation of moderate‐to‐severe dementia.

In summary, future work should refine remote protocols, incorporate information and clinical covariates, and validate performance across more diverse populations and the full spectrum of disease severity to improve precision and generalizability.

## CONSENT

The study involved secondary analysis of existing, de‐identified data from the Knight ADRC MAP. All participants have previously provided informed consent for participation and data use. No additional consent was required for this analysis.

## CONFLICT OF INTEREST STATEMENT

The authors declare no conflicts of interest. Author disclosures are available in the Supporting Information.

## Supporting information



Supporting Information: alz71601‐sup‐0001‐ICMJE.pdf

Supporting Information: alz71601‐sup‐0001‐Table 1.pdf
