# Supplementary material for: Evaluating the accuracy and reliability of telephone‐derived Clinical Dementia Rating scores: A comparative analysis with in‐person assessments
Source: Alzheimers Dement. 2026 Jun 18;22(6):e71601. doi: 10.1002/alz.71601 (PMC13279448; doi:10.1002/alz.71601)
Supplement: Supplementary file 2 — Supporting Information: alz71601‐sup‐0001‐Table 1.pdf [file ALZ-22-e71601-s001.pdf]

Analysis	Weighted Kappa	95% CI Lower	95% CI Upper
One Pair Per Participant	0.54	0.45	0.62
Stable Subset	0.48	0.34	0.62
<=3 months	0.53	0.47	0.59
7-12 months	0.49	0.40	0.59
Sex:F	0.49	0.41	0.58
Sex:M	0.55	0.47	0.64
Race: Black	0.60	0.45	0.76
Race: White	0.51	0.44	0.57
Education:<16 years	0.49	0.40	0.59
Education:>=16 years	0.54	0.46	0.62

Supplementary Table 1: Sensitivity and Subgroup Analysis
